# Angiogenesis-Related Biomarkers in Patients with Alcoholic Liver Disease: Their Association with Liver Disease Complications and Outcome

**DOI:** 10.1155/2014/673032

**Published:** 2014-05-18

**Authors:** Beata Kasztelan-Szczerbinska, Agata Surdacka, Maria Slomka, Jacek Rolinski, Krzysztof Celinski, Halina Cichoz-Lach, Agnieszka Madro, Mariusz Szczerbinski

**Affiliations:** ^1^Department of Gastroenterology with Endoscopy Unit, Medical University of Lublin, 8 Jaczewski Street, 20-954 Lublin, Poland; ^2^Department of Clinical Immunology, Medical University of Lublin, 4A Chodzki Street, 20-093 Lublin, Poland

## Abstract

Angiogenesis is believed to be implicated in the pathogenesis of alcoholic liver disease (ALD). We aimed to explore the usefulness and accuracy of plasma angiogenic biomarkers for noninvasive evaluation of the severity of liver failure and ALD outcome. One hundred and forty-seven patients with ALD were prospectively enrolled and assessed based on their (1) gender, (2) age, (3) severity of liver dysfunction according to the Child-Turcotte-Pugh and MELD scores, and (4) the presence of ALD complications. Plasma levels of vascular endothelial growth factor (VEGF-A) and angiopoietins 1 and 2 (Ang1 and Ang2) were investigated using ELISAs. Multivariable logistic regression was applied in order to select independent predictors of advanced liver dysfunction and the disease complications. Significantly higher concentrations of Ang2 and VEGF-A in ALD patients as compared to controls were found. There was no difference in Ang1 levels in both groups. A positive correlation of Ang2 levels with INR (Rho 0.66; *P* < 0.0001) and its inverse correlation with plasma albumin levels (Rho –0.62; *P* < 0.0001) were found. High Ang2 concentrations turned out to be an independent predictor of severe liver dysfunction, as well as hepatic encephalopathy and renal impairment. Ang2 possessed the highest diagnostic and prognostic potential among three studied angiogenesis-related molecules.

## 1. Introduction

Alcoholic liver disease (ALD) with subsequent progression to cirrhosis and hepatocellular carcinoma (HCC) has become a predominant liver disease in Europe [1]. Excessive ethanol consumption induces chronic inflammation in liver parenchyma and triggers both fibrogenesis and an angiogenic response [2]. Angiogenesis appears to be a major contributor to the development of liver complications including portal hypertension, portal-systemic collaterals, and hyperdynamic splanchnic circulation. On the other hand, intrahepatic vascular remodelling with capillarization of sinusoids and alcohol-related central zone steatosis of lobuli alter hepatic oxygen supply leading to hypoxia with formation of new vessels and finally create a vicious circle [3]. Angiogenesis initially may act as a defense mechanism preventing ischaemic damage and facilitating an access of immune cells to the sites of injury. However, neovascularization often results in an immature leaky vascular network, structurally and functionally different than normal. As a consequence, it may cause tissue deterioration and further aggravate inflammation. Accumulating scientific evidence suggests that there is a close relationship between angiogenesis and inflammation, which parallel each other and may favor liver disease progression [4–9].

Angiogenesis is regulated by a balance between pro- and antiangiogenic factors in a mutually dependent manner. It is crucial for the maintenance of normal structure and function of the human body vasculature [10]. Imbalance in secretion of angiostatic molecules leads to the so-called “angiogenic switch” that initiates the formation of new blood vessels [11].

Vascular endothelial growth factor (VEGF) is best known as the most potent stimulator of both normal and pathological angiogenesis. In the postnatal period, it preserves integrity of endothelium and acts as a mitogen for endothelial cells. VEGF is a potent mediator in wound healing, and it also induces vascular permeability inside injured tissues.

The release of VEGF increases under hypoxic conditions. Its expression is regulated by the hypoxia inducible factor (HIF-1a), which triggers VEGF transcription [12]. This fact indicates that VEGF participates in the early stage of angiogenesis. As a result the transition of endothelial cells from an idle to active state together with their proliferation, migration, and formation of new vessels may occur.

Tyrosine kinase receptors (Tie1 and Tie2) and their ligands angiopoietins 1–4 (Ang1, -2, -3, and -4) play a key role during the late phase of angiogenesis and are responsible for the maturation of newly formed vascular structures. The best described and characterized are two angiopoietins: Ang1 and Ang2. The activity of the angiopoietin/Tie system determines the stabilization of new vessels. Both Ang1 and Ang2 interact with the same site of the Tie2 receptor having similar affinity toward it, but only Ang1 induces its phosphorylation and subsequent activation [13].

There is growing evidence that the angiopoietin/Tie signaling may influence the evolution of inflammation [14]. Ang1 appears to be a potent Tie2 activator, as well as a regulator of blood vessel formation and maturation. Experimental studies have demonstrated that Ang1 acts as an anti-inflammatory molecule [15], but on the other hand it may induce significant complications such as pulmonary hypertension [16]. It was found that Ang1 protected against endotoxemia during shock and diminished microvascular leakage [17]. Ang1 also neutralizes an activity of the tissue factor (TF) relevant to the induction of coagulation, thrombosis, and inflammatory response. Furthermore, Ang1 reduces VEGF-related adhesion of leukocytes to endothelium [18, 19].

In contrast, Ang2 acts as a competitive antagonist of Ang1 and downregulates Tie2 signaling [13]. It exerts proinflammatory effects [19–22]. High plasma concentrations of Ang2 were described in psoriasis and inflammatory bowel diseases [23]. Data obtained from animal models indicated that its deficiency protected mice against lethal inflammatory activation induced by* Staphylococcus aureus* in the course of peritonitis [24]. In the clinical setting of sepsis, Ang2 serum concentrations may increase up to 20-fold and its high levels were associated with worse survival of patients [25, 26]. Furthermore, significantly elevated serum levels of Ang2 were observed during tumorigenesis in patients with the squamous cell carcinoma of the esophagus [27], liver cancer [28], and lung cancer [29]. Unfortunately, many pathways crucial for the promotion of pathological angiogenesis and activated by various stimuli are not completely explained yet.

On this background, we have designed a study in order to assess concentrations of selected molecules of angiogenesis, that is, vascular endothelial growth factor (VEGF-A) and angiopoietins 1 and 2 (Ang1 and Ang2) in peripheral blood of patients with ALD in comparison to healthy controls (HC). We hypothesized that their synthesis might be increased during the course of ALD as a consequence of the local and systemic inflammatory response and have an impact on the disease deterioration and the development of complications. We further aimed to investigate the usefulness and accuracy of selected angiogenic biomarkers in the noninvasive evaluation of the degree of liver failure and ALD outcome.

## 2. Material and Methods

The study cohort has been described in detail in our previously published report concerning the adipokine assessment [30].

Briefly, 147 consecutive adult inpatients (pts) with ALD, admitted to the Department of Gastroenterology with Endoscopy Unit in Lublin, were prospectively enrolled over a 2-year period and followed for 90 days. Thirty matching volunteers, who pledged abstinence or alcohol consumption as no more than 20 g ethanol per day, served as a control group.

The ALD diagnosis was established based on typical symptoms and physical findings of chronic liver disease, high aminotransferase levels, AST/ALT ratio above 2, and imaging studies in the setting of excessive alcohol intake. Alcohol abuse was confirmed by the AUDIT-C (Alcohol Use Disorders Identification Test-Consumption) questionnaire [31]. The positive result of AUDIT-C, in addition to the amount of alcohol intake, was an inclusion criterion. The daily alcohol intake in the ALD group ranged as follows: in females 40 g/d to more than 100 g/d and in males 50 g/d to more than 100 g/d.

According to the study protocol, eligible patients signed the informed consent, completed their medical history, and answered the AUDIT-C questionnaire prior to the investigation.

Neither corticosteroids nor pentoxifylline were administered to any individual at the time of enrollment. Demographic data as well as all procedures were recorded and performed within 48 hours after hospital admission. Blood samples were collected at 07:30 AM after a minimum 8-hour overnight fast. Other cofactors of chronic liver injury were excluded.

The severity of liver failure at baseline was established using the Child-Turcotte-Pugh (CTP) [32] and the model of end-stage liver disease (MELD) [33] criteria. The calculators available on the internet (i.e., http://www.mayoclinic.org and http://potts-uk.com/livercalculator.html) were adopted to calculate both scores.

Patients were included into different subgroups according to their

1. gender,
2. age,
3. the severity of liver dysfunction according to the CTP (classes A, B, and C) and MELD (≥20 or <20) scores,
4. the presence of ALD complications at the time of hospital admission, that is, ascites, hepatic encephalopathy (HE), oesophageal varices, cholestasis, and renal impairment.

Subjects with severe comorbidities present at the time of enrollment, that is, malignancy, pulmonary insufficiency, heart failure, and uncontrolled diabetes, were excluded.

Cholestasis was defined in accordance with the recommendations of the European Association for the Study of the Liver (EASL), that is, alkaline phosphatase (AP) greater than 1.5 times above the upper limit of normal (ULN) and the activity of *γ*-glutamyl transpeptidase (GGT) more than three times the ULN [34]. Abdominal ultrasonography was performed to confirm the presence of ascites and to exclude other causes of cholestasis. The presence of mitochondrial antibodies (AMA) and drugs hepatotoxicity were also ruled out.

The presence of esophageal varices was confirmed endoscopically.

The level of serum creatinine above 1.3 mg/dL (the upper limit of normal) was considered a criterion of renal impairment.

Only individuals for whom all the required laboratory data were available at admission were included in the trial.

All enrolled patients were inpatients at the starting point of the study. They were discharged from the hospital once ethanol withdrawal symptoms have disappeared, complications of liver failure (i.e., coagulopathy, jaundice, encephalopathy, etc.) have resolved, and liver function has begun to improve. Subsequent follow-up visits with their examination during next 90 days were set at least once a month (generally every 2 weeks) in the liver clinic or during any hospital admission if required. Two of the nonsurvivors returned to our department after their condition worsened and they died in the hospital. The majority of nonsurvivors (10 out of 12) were treated continuously without any hospital discharge.

We used enzyme-linked immunosorbent assays to measure concentrations of selected angiogenic biomarkers, that is, vascular endothelial growth factor-A (VEGF-A) and angiopoietins 1 and 2 (Ang1 and Ang2) with commercially available kits (Quantikine ELISA kit, R&D Systems, USA). Blood samples were obtained by venipuncture into vacutainer tubes containing EDTA and centrifuged within 30 minutes for 15 minutes at 4°C. As recommended by the R&D Systems instructions, an additional centrifugation step was performed for complete platelets removal. Plasma samples were stored frozen at ≤−20°C until the time of examination.

The examination was conducted according to the procedure recommended by the producer and described in the attached materials. Measurements were performed using VictorTM3 Reader (PerkinElmer, USA).

The study protocol conforms to the ethical guidelines of the 1975 Declaration of Helsinki (6th revision, 2008) as reflected in a priori approval by the institutional review board of Medical University of Lublin.

## 3. Statistical Analysis

Statistical analysis was performed using the Statistica 10 software package (StatSoft, Poland). The distribution of the data in the groups was preliminarily evaluated by Kolmogorov-Smirnov test. A skewed distribution of checked values was found, so continuous variables were presented as medians with interquartile range and assessed using Mann-Whitney* U* test. Categorical variables were described as numbers with percentage and compared using either Fisher's exact test or the *χ* ^2^ test as appropriate. The differences in angiogenic molecules levels between CTP classes were analyzed using Kruskal-Wallis and multiple comparisons post hoc tests. Spearman's rank correlation test was used for the assessment of association between parameters of liver function, traditional indicators of inflammation, and biomarkers plasma levels. The receiver operating curves (ROC) for significant angiogenic biomarkers were constructed and their areas under the curve (AUCs) were checked in order to assess their accuracy in predicting the degree of liver failure and the development of ALD complications. The method of DeLong et al. [35] for the calculation of the standard error of the AUC was used. The Youden index and its associated cutoff point were estimated for each marker [36]. Moreover, multivariable logistic regression was applied to select independent predictors of serious liver dysfunction and the development of ALD complications. A two-sided *P* value of less than 0.05 was considered to be associated with statistical significance.

## 4. Results

One hundred and forty-seven patients (pts) met the inclusion criteria, 107 males (72.8%) and 40 females (27.2%). Their mean age was 49.84 ± 11.53 and 48.82 ± 9.94, respectively.

Of the 147 pts with ALD, 12 (8.16%) died from complications of liver failure within 90 days of followup. The matching control group consisted of 17 (56.7%) males and 13 (43.3%) females aged 44.31 ± 10.23 and 43.11 ± 8.43, respectively. The baseline characteristics of ALD patients are presented in Table 1.

At the beginning, we measured plasma concentrations of three angiogenic molecules in patients (pts) with ALD and healthy controls (HC). While Ang2 and VEGF-A levels were found significantly increased in the ALD group, Ang1 concentrations did not differ in comparison with HC. Moreover, the Ang2/Ang1 ratio was significantly higher in ALD pts (median; 25–75 interquartile range: 1.97; 0.61–9.80 versus 0.91; 0.39–1.28, resp.; *P* = 0.002) and inversely correlated with VEGF-A concentration (Rho −0.54; 95% CI −0.65 to −0.42; *P* < 0.0001). The results are presented in Table 2.

We observed no significant differences in plasma concentrations of angiogenesis-related markers between both sexes either in the control or in the ALD group (*P* > 0.05).

Since recently published data have shown that age-related alterations may have an influence on angiogenesis [27, 37], the next step of our study included the assessment of plasma concentrations of angiogenic biomarkers in two age subgroups: ≥50 and <50 years old. As expected, significant differences in the biomarker plasma levels were found in both subgroups. The results are presented in Table 3.

Our subsequent analysis revealed that of three angiogenic molecules only the level of Ang2 rose significantly with the severity of liver dysfunction classified according to the CTP as well as MELD scores. The results are shown in Table 4.

**Figure 1:**
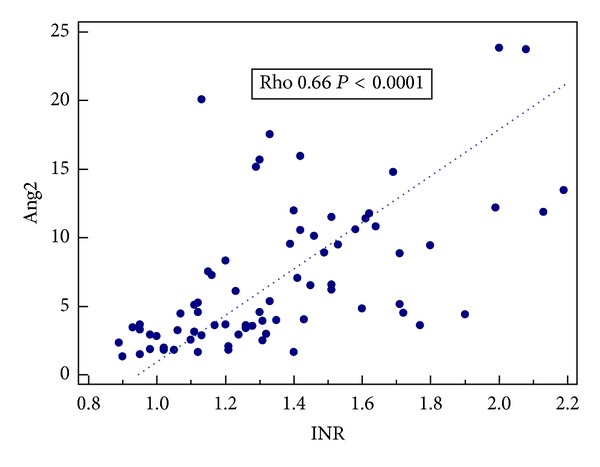
Correlation between Ang2 concentrations (ng/mL) and INR.

**Figure 2:**
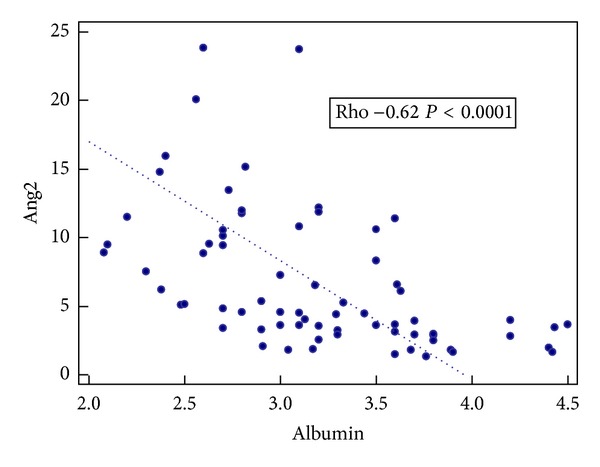
Correlation between Ang2 concentrations (ng/mL) and serum albumin levels (g/dL).

Further analyses concerning liver function parameters showed a positive correlation of plasma Ang1 concentrations with liver enzymes (ALT: Rho 0.28; *P* = 0.02; AST: Rho 0.28; *P* = 0.01; AP: Rho 0.30; *P* = 0.01; GGT: Rho 0.41; *P* = 0.0004). Moreover a significant correlation of Ang2 concentrations with two major liver function parameters, that is, INR and plasma albumin levels, was also found (Rho 0.66; *P* < 0.0001; Rho −0.62; *P* < 0.0001, resp.). Results are presented in Figures 1 and 2.

The studied angiogenic biomarkers weakly correlated with traditional indicators of inflammation: the white blood cell count (Ang1: Rho 0.25; *P* = 0.002; Ang2: Rho 0.22; *P* = 0.007; VEGF-A: Rho 0.25; *P* = 0.002) and CRP level (Ang2: Rho 0.26; *P* = 0.002; VEGF-A: Rho 0.22; *P* = 0.007).

The next step of our investigation was focused on exploration of a possible association between ALD complications and plasma angiogenic biomarker concentrations.

For angiogenic biomarkers, in which plasma levels were significantly different in subgroups of subjects selected according to the severity of liver dysfunction (MELD ≥ 20) and the presence of ALD complications, the areas under the curve (AUCs) were assessed and their diagnostic accuracy was compared. The results revealed that Ang2 possessed the highest diagnostic and prognostic potential among studied angiogenesis-related molecules. The results are summarized in Tables 5, 6, and 7.

## 5. Discussion

Our study represents an in-depth evaluation of the usefulness of angiogenesis-related biomarkers in noninvasive monitoring of the ALD course. It may provide insights into methods of preventing the ALD progression and the development of its complications. Despite intensive research, the role of Ang1, Ang2, and VEGF in the evolution of liver disease remains unclear. To our knowledge, this is the first study which has demonstrated the association of systemic concentrations of Ang2 and VEGF-A with ALD evolution. Nowadays, it is increasingly evident that the liver disease etiology is linked to the dynamics of chronic wound healing process, the development of fibrosis, and the rate of progression to cirrhosis [38]. In this context, we limited our investigations to the homogeneous group of patients with ALD.

The pivotal finding of the study is the identification of Ang2 as an independent predictor of advanced liver dysfunction as well as two major ALD complications (i.e., HE and renal impairment). We found that high baseline plasma Ang2 levels were related to the poor disease outcome and its more aggressive course.

We also observed that levels of circulating Ang1 in healthy adults were higher than Ang2 and the Ang2/Ang1 ratio was significantly lower compared to patients with ALD (median; interquartile range: 0.91; 0.35–1.59 versus 1.97; 0.61–9.80; *P* = 0.002). This fact may reflect a defense mechanism against the potentially harmful effects of Ang2. Ang2 is believed to endure an impact of Ang1 and induce vascular destabilization [39, 40].

Previous experimental results showed the synergistic interaction between inflammation and angiogenesis in the healing of damaged tissues [41, 42].

On the other hand, angiogenesis generated at the early stage of liver disease may promote the transition of acute into a chronic phase of inflammation. It is also possible that a debilitating immune defect might prevent damping of inflammation in a subgroup of ALD patients. Our study revealed the close relationship between angiogenesis and inflammation in the course of ALD. We found a positive correlation between the levels of three angiogenic molecules and traditional markers of inflammation (i.e., white cells count and CRP level). Our results are consistent with previous reports concerning the similar topic [43, 44]. Further explanation of the mutual dependence of both processes in the course of ALD raises hope for its effective future treatment. At present, preliminary trials of simultaneous modulation of inflammation and angiogenesis are being conducted in other inflammatory diseases [45–47].

In the ALD group, Ang2 and VEGF-A concentrations were significantly higher in comparison to healthy individuals regardless of their gender (Table 2). Also, as mentioned above, the Ang2/Ang1 ratio was significantly higher in this group and inversely correlated with VEGF-A concentration. Plasma concentrations of Ang2 and VEGF-A in ALD subgroup were significantly different in comparison to controls and depended on the grade of liver dysfunction classified according to the CTP and MELD scores. Ang2 concentrations significantly increased in parallel with the severity of liver failure assessed by both the CTP and MELD scores. In contrast, VEGF-A plasma levels showed a tendency (*P* = 0.07) to lower values in subgroups with advanced stages of liver dysfunction classified by the CTP criteria (Table 4).

These results were in agreement with the results obtained from the next analysis, that is, correlation tests. A significant correlation of Ang2 levels with synthetic liver function parameters was found: negative for the albumin level and positive for INR. Our results suggest that Ang2 may be a relevant biomarker of liver function impairment in ALD pts and indicate the potential for its use in clinical practice.

On the other hand, Ang1 and VEGF-A plasma concentrations showed a positive correlation with cholestatic enzymes (AP and GGT). The levels of both molecules were significantly higher in the subset of pts with signs of cholestasis (Table 5). In addition, Ang1 concentrations showed a positive correlation with aminotransferase levels.

Further evaluation showed a different association of Ang2 and VEGF-A plasma levels with the development of ALD complications. Ang2 concentrations were significantly higher, but VEGF-A was significantly lower in patients with ascites, hepatic encephalopathy (HE), and esophageal varices (Table 5). In addition, increased levels of Ang2 were found in the subgroup with renal impairment as well as in nonsurvivors (Table 5). The results obtained in our study are in line with findings of other authors who also reported that angiogenic factors imbalance might influence the development of complications in different diseases, for example, in diabetes [48, 49].

It is likely that the development of ALD complications may result from endotoxemia which is quite frequent phenomenon in the course of the disease. It has been found that endotoxemia causes microcirculatory endothelial dysfunction with increased vessel permeability and can lead to a disintegration of vascular system together with organ failure [50, 51]. Moreover, previously published reports indicated that administration of an Ang1 variant reduced the endotoxin-related vascular leak by restoring cell tight junctions and by decline in leukocyte infiltration [52]. Therefore the significantly lower Ang2/Ang1 ratio observed in our controls in comparison to the ALD group seems to create a physiological defense mechanism in order to keep vascular stability. Perhaps the protective effect of Ang1 substitution could be used in a future ALD therapy. Similar attempts to prevent other organs dysfunction (e.g., kidney and lung) have been ongoing for several years [53–55].

The results of multivariate logistic regression confirmed the independent impact of Ang2 on the severity of liver failure (MELD ≥ 20) and the development of two major ALD complications, that is, HE and renal impairment (Table 7). Our results suggest that Ang2 may play a key role in the progression of ALD and be a valuable diagnostic as well as prognostic indicator for this group of patients. Other authors reported that an increased expression of Ang2 was observed also in other inflammatory and/or neoplastic disorders such as sepsis [56], colorectal and liver cancer [57, 58], and stomach cancer [59]. Helfrich et al. [60] observed an association of elevated Ang2 levels with the disease progression and survival in patients with melanoma. Also Detjen et al. [61] found a positive correlation of Ang2 blood concentrations with tumor spread and survival in neuroendocrine tumors.

Although many aspects of Ang2 action are still unclear, the observations obtained from experimental models and the results of clinical studies provide an insight into the potential utility of Ang2 as a prognostic predictor in a variety of disorders associated with active vascular remodeling; ALD could be one of them as suggested by our study.

Limitations of the present study come from the relatively small sample size. It was a single-center trial so it should be emphasized that the results before their wide application require being confirmed in future multicenter trials. Such validation may help to avoid possible errors resulting from research techniques or subjective differences in the patient population selection.

If the results are confirmed, two potential prospects emerge from our study.

1. Selected proangiogenic molecules may serve as an easy noninvasive diagnostic tool in the ALD evaluation.
2. The antiangiogenic therapy seems to be able to modulate the disease progression and therefore requires further detailed investigations.

Development of tools and strategies to limit the simultaneous chronic inflammatory response and angiogenesis in the course of ALD may help to avoid their subsequent adverse effects and prevent liver failure and diminish the need for liver transplantation.

## Figures and Tables

**Table 1:** Basic characteristics of patients with ALD based on their gender*.

	ALD group (*n* = 147)						*P*
	Females (*n* = 40)			Males (*n* = 107)			
	Median	95% CI	25–75 P	Median	95% CI	25–75 P	
Age years	51.00	48.03–54.96	45.00–56.00	51.00	48.00–52.49	40.00–60.00	0.19

ALT IU/L	39.50	28.03–44.93	23.00–47.00	56.00	50.00–69.00	35.25–84.00	**0.004**

ASP IU/L	100.50	78.45–114.90	66.00–120.00	110.00	78.51–131.00	64.50–189.00	0.72

AP IU/L	118.50	111.68–156.27	105.00–179.00	129.00	118.00–148.00	79.00–223.00	0.62

GGT IU/L	415.00	174.00–543.00	172.00–772.00	359.00	200.50–504.88	93.00–1066.00	**0.020**

T-Bil mg/dL	4.20	3.51–5.27	3.30–8.40	3.00	1.75–4.00	1.10–8.10	0.70

Alb g/dL	3.10	2.70–3.29	2.63–3.50	3.20	3.00–3.30	2.73–3.61	0.12

INR	1.45	1.39–1.64	1.31–1.71	1.21	1.16–1.30	1.07–1.43	**0.034**

Crea mg/dL	0.80	0.70–0.80	0.70–1.00	0.90	0.90–1.00	0.80–1.10	0.51

Na mEq/L	139.00	136.03–140.96	134.00–141.00	138.00	136.51–139.00	134.00–140.00	0.38

Hgb g/dL	11.20	10.34–11.50	9.70–12.00	12.10	11.60–12.70	10.30–13.50	**<0.001**

RBC ×10^6^ kom/uL	3.17	3.08–3.50	2.86–3.52	3.86	3.57–3.97	3.15–4.11	**<0.001**

PLT ×10^3^ kom/uL	135.50	114.38–137.96	97.00–251.00	136.00	116.00–166.46	80.00–202.00	0.81

WBC ×10^3^ kom/uL	8.12	5.42–11.63	4.89–13.04	7.12	6.30–8.28	5.01–10.80	0.75

NEUT ×10^3^ kom/uL	8.44	3.20–8.97	2.57–13.51	5.02	4.19–6.10	2.91–7.92	**0.053**

NLR	4.38	2.34–4.52	2.34–7.63	3.47	3.26–4.45	2.13–6.04	0.12

CRP mg/L	17.33	16.19–33.14	5.98–42.17	17.53	13.40–21.30	5.01–43.00	0.58

mDF	17.35	12.00–22.96	9.00–28.00	9.00	6.00–12.00	4.00–16.74	0.21

MELD	17.50	15.03–18.00	12.00–20.00	15.00	14.00–16.00	11.00–17.00	**0.047**

CTP	9.50	9.00–10.00	8.00–10.00	7.00	7.00–8.00	7.00–9.00	**<0.001**

*Alb: albumin (normal range (NR) 3.2–4.8); ALT: alanine aminotransferase ((NR) < 31); AP: alkaline phosphatase (NR 45–129); AST: aspartate aminotransferase (NR < 34); Crea: creatinine (NR 0.5–1.1); CRP: C-reactive protein (NR 0.0–5.0); CTP: Child-Turcotte-Pugh score; GGT: gamma-glutamyl transpeptidase (NR < 50.0); Hgb: hemoglobin (NR 14.0–18.0); INR: international normalized ratio (NR 0.8–1.2); MELD: model for end-stage liver disease; Na: sodium (NR 136–145); NEUT: neutrophils (NR 1.8–7.7); NLR: neutrophil to lymphocyte ratio; PLT: platelets (NR 130–400); 25–75 P: percentiles; RBC: red blood cells (NR 4.5–6.1); T-Bil: total bilirubin (NR 0.3–1.2); WBC: white blood cells (NR 4.8–10.8).

**Table 2:** Comparison of plasma angiogenesis-related biomarkers in ALD patients and the control group*.

	Biomarkers of angiogenesis						*P*
	ALD patients (*n* = 147)			Controls (*n* = 30)			
	Median	95% CI	25–75 P	Median	95% CI	25–75 P	
Ang1 ng/mL	2.90	2.09–3.26	0.99–5.28	3.02	1.46–4.97	1.06–6.12	0.83

Ang2 ng/mL	4.58	3.99–6.09	3.12–9.97	1.95	1.33–2.30	1.10–2.43	**<0.0001**

VEGF-A pg/mL	85.27	68.73–99.16	40.01–207.91	48.47	36.58–67.30	31.71–70.95	**0.001**

*Ang1, Ang2: angiopoietins 1 and 2; CI: confidence interval; P: percentile; VEGF: vascular endothelial growth factor.

**Table 3:** Comparison of plasma angiogenic biomarkers according to the age of patients with ALD.

	Angiogenesis-related biomarkers in ALD group						*P*
	Age < 50 (*n* = 64)			Age ≥ 50 (*n* = 83)			
	Median	95% CI	25–75 P	Median	95% CI	25–75 P	
Ang1 ng/mL	3.69	2.32–5.14	1.16–8.57	2.09	1.69–3.02	0.16–4.67	**0.017**

Ang2 ng/mL	3.99	3.58–4.83	2.49–6.21	7.23	4.46–9.48	3.36–11.56	**0.001**

VEGF-A pg/mL	100.22	67.62–231.94	53.02–369.57	73.79	61.40–89.03	30.60–145.22	**0.054**

**Table:** (a) CTP class

	Class A (*n* = 30)			Class B (*n* = 73)			Class C (*n* = 44)			*P*
	Median	95% CI	25–75 P	Median	95% CI	25–75 P	Median	95% CI	25–75 P	
Ang1 ng/mL	2.96	1.81–3.26	1.16–5.06	3.17	1.98–4.72	1.03–6.20	2.08	1.02–3.66	0.00–4.65	0.28

Ang2 ng/mL	2.94	2.35–3.85	1.88–3.99	4.56	3.57–5.35	2.91–7.23	10.32	9.42–11.86	5.12–13.44	**<0.0001**

VEGF-A pg/mL	100.13	73.50–120.66	62.57–231.66	89.21	61.40–203.16	30.60–358.15	67.21	48.27–81.25	33.74–112.68	0.07

**Table:** (b) MELD score

	<20 points (*n* = 117)			≥20 points (*n* = 30)			*P*
	Median	95% CI	25–75 P	Median	95% CI	25–75 P	
Ang1 ng/mL	2.90	2.09–3.36	1.03–5.84	2.71	0.11–3.72	0.00–5.1	0.16

Ang2 ng/mL	3.90	3.56–4.56	2.86–7.72	11.37	7.05–13.19	5.12–15.94	**<0.0001**

VEGF-A pg/mL	86.55	71.81–100.18	43.10–207.91	64.60	27.11–148.12	23.25–246.83	0.20

**Table 5:** Plasma angiogenesis-related biomarkers in subgroups of ALD patients according to the presence of the disease complications.

	Ascites						*P*
	Absent (*n* = 58)			Present (*n* = 89)			
	Median	95% CI	25–75 P	Median	95% CI	25–75 P	
Ang1 ng/mL	3.19	2.32–5.04	1.51–5.29	2.09	1.50–3.17	0.16–4.82	0.11

Ang2 ng/mL	3.21	2.52–3.88	1.88–4.58	7.53	5.35–9.43	3.89–11.40	**<0.0001**

VEGF-A pg/mL	100.22	82.63–196.53	61.40–333.36	68.73	58.66–86.55	29.43–149.34	**0.031**

	Hepatic encephalopathy						
	Absent (*n* = 127)			Present (*n* = 20)			*P*
	Median	95% CI	25–75 P	Median	95% CI	25–75 P	

Ang1 ng/mL	2.96	2.09–3.28	1.03–5.70	1.77	0.00–3.64	0.00–3.69	0.07

Ang2 ng/mL	4.45	3.64–5.26	2.92–8.84	10.13	5.61–14.87	4.83–15.16	**0.0009**

VEGF-A pg/mL	91.79	71.66–115.74	44.30–243.11	49.17	25.50–84.63	25.46–85.27	**0.003**

	Oesophageal varices						
	Absent (*n* = 60)			Present (*n* = 87)			*P*
	Median	95% CI	25–75 P	Median	95% CI	25–75 P	

Ang1 ng/mL	3.06	2.33–5.14	1.75–7.28	2.32	1.51–3.29	0.16–4.70	0.08

Ang2 ng/mL	3.63	2.89–5.26	1.88–6.21	5.12	4.37–8.31	3.55–11.46	**0.001**

VEGF-A pg/mL	100.22	72.73–231.94	61.98–358.65	71.66	52.43–89.51	26.62–146.57	**0.005**

	Cholestasis						
	Absent (*n* = 117)			Present (*n* = 30)			*P*
	Median	95% CI	25–75 P	Median	95% CI	25–75 P	

Ang1 ng/mL	2.32	1.51–3.05	0.49–5.08	4.72	3.28–6.40	2.90–7.28	**0.002**

Ang2 ng/mL	4.83	3.64–6.55	2.90–10.52	3.90	3.39–4.55	3.12–6.21	0.28

VEGF-A pg/mL	71.81	61.40–88.396	33.01–168.11	121.73	101.30–244.22	81.52–402.69	**0.001**

	Renal impairment						
	Creatinine < 1.3 mg/dL (*n* = 125)			Creatinine ≥ 1.3 mg/dL (*n* = 22)			*P*
	Median	95% CI	25–75 P	Median	95% CI	25–75 P	

Ang1 ng/mL	2.32	1.69–3.18	0.65–5.29	3.27	3.05–5.14	2.71–5.28	0.08

Ang2 ng/mL	4.45	3.63–5.12	2.92–9.06	9.42	7.13–11.40	5.09–11.99	**0.004**

VEGF-A pg/mL	73.79	66.81–91.62	33.74–231.66	112.68	85.52–158.08	64.60–207.91	0.32

	Disease outcome						
	Survivors (*n* = 135)			Nonsurvivors (*n* = 12)			*P*
	Median	95% CI	25–75 P	Median	95% CI	25–75 P	

Ang1 ng/mL	3.05	1.98–3.52	1.00–5.70	2.28	0.00–2.71	0.00–2.71	0.10

Ang2 ng/mL	4.51	3.80–5.26	2.92–8.92	11.74	9.48–13.44	9.48–13.44	**0.001**

VEGF-A pg/mL	85.270	68.73–95.69	40.84–207.31	73.14	25.46–246.83	25.46–246.83	0.48

**Table 6:** Comparison of the diagnostic accuracy (AUC) of single variables in the diagnosis of advanced liver dysfunction (MELD ≥ 20) and ALD complications (univariable analysis)*.

Complication of ALD	Variable	*P* value	AUC (95% CI)	SE
MELD ≥ 20	**Ang2**	<0.0001	0.829 (0.758–0.886)	0.036
	CRP	0.004	0.609 (0.527–0.686)	0.058
	RBC	0.003	0.675 (0.596–0.747)	0.055
	WBC	0.0003	0.656 (0.577–0.730)	0.061
	Ascites	0.003	0.652 (0.572–0.725)	0.050
	HE	<0.0001	0.666 (0.587–0.739)	0.058

Ascites	**Ang2**	<0.0001	0.772 (0.695–0.837)	0.041
	Albumin	<0.0001	0.819 (0.748–0.877)	0.036
	ALT	0.0001	0.710 (0.633–0.779)	0.041
	AST	0.003	0.606 (0.526–0.683)	0.047
	INR	<0.0001	0.808 (0.739–0.866)	0.036
	RBC	0.002	0.663 (0.584–0.736)	0.044
	WBC	0.008	0.597 (0.517–0.674)	0.045

HE	**Ang2**	0.0001	0.731 (0.652–0.801)	0.063
	**VEGF-A**	0.022	0.705 (0.624–0.777)	0.050
	AP	0.006	0.652 (0.567–0.730)	0.071
	Albumin	0.005	0.686 (0.605–0.759)	0.055
	T-bilirubin	0.0001	0.770 (0.697–0.833)	0.048
	INR	0.0001	0.737 (0.661–0.804)	0.059
	PLT	0.035	0.633 (0.553–0.708)	0.057
	Ascites	0.012	0.646 (0.567–0.720)	0.056

Renal impairment (crea > 1.3 mg/dL)	**Ang2**	0.003	0.692 (0.610–0.765)	0.062
	Albumin	0.034	0.654 (0.572–0.729)	0.059
	AST	0.042	0.601 (0.521–0.678)	0.062
	AP	0.030	0.677 (0.593–0.753)	0.068
	Na	0.012	0.588 (0.508–0.666)	0.080
	CRP	0.001	0.714 (0.636–0.784)	0.060
	WBC	0.011	0.689 (0.611–0.760)	0.053
	RBC	0.031	0.688 (0.610–0.759)	0.059

Poor outcome (nonsurvival)	**Ang2**	0.009	0.788 (0.713–0.851)	0.059
	Bilirubin	0.0004	0.765 (0.691–0.828)	0.059
	Albumin	0.0004	0.818 (0.747–0.876)	0.061
	Na	0.003	0.751 (0.676–0.816)	0.081
	AP	0.024	0.641 (0.556–0.720)	0.102
	INR	0.009	0.735 (0.659–0.802)	0.057
	HE	0.005	0.652 (0.573–0.726)	0.086

*AUC: area under the ROC curve; CI: confidence interval; SE: standard error.

**Table 7:** Independent predictors of advanced liver dysfunction (MELD ≥ 20) and ALD complications (multivariable analysis)*.

Complication of ALD	Variable	*P* value	Adjusted OR (95% CI)	AUC (95% CI)	SE
MELD ≥ 20	**Ang2**	<0.0001	1.358 (1.190–1.550)	0.908 (0.844–0.952)	0.028
	HE	0.021	4.796 (1.269–18.124)		

HE	**Ang2**	0.010	1.256 (1.057–1.493)	0.934 (0.876–0.970)	0.029
	**VEGF-A**	0.066	0.977 (0.953–1.002)		
	AP	0.035	1.016 (1.001–1.032)		
	T-bilirubin	0.024	1.126 (1.015–1.249)		
	PLT	0.014	0.980 (0.965–0.996)		

Renal impairment (crea > 1.3 mg/dL)	**Ang2**	0.009	1.128 (1.030–1.236)	0.775 (0.695–0.842)	0.060
	CRP	0.006	1.023 (1.006–1.039)		

*AUC: area under the ROC curve; CI: confidence interval; Crea: creatinine; HE: hepatic encephalopathy; OR: odds ratio; SE: standard error.
